# Implementation of iPads to Increase Compliance With Delivery of New Parent Education in the Mother–Baby Unit: Retrospective Study

**DOI:** 10.2196/18830

**Published:** 2021-06-15

**Authors:** Haritha Pavuluri, Alicia Grant, Alexander Hartman, Lauren Fowler, Jennifer Hudson, Patrick Springhart, Ann Blair Kennedy

**Affiliations:** 1 University of South Carolina School of Medicine Greenville Greenville, SC United States; 2 School of Medicine, Department of Pediatrics University of California, San Francisco San Francisco, CA United States; 3 Department of Pediatrics Prisma Health-Upstate Greenville, SC United States; 4 Department of Urology Prisma Health-Upstate Greenville, SC United States

**Keywords:** technology, handheld computers, workflow, education, newborn, head trauma

## Abstract

**Background:**

Abusive head trauma (AHT) is a serious health problem affecting more than 3000 infants annually in the United States. The American Academy of Pediatrics and the Centers for Disease Control and Prevention (CDC) recommend that health care providers counsel new parents about the dangers of AHT. Previous studies demonstrate that parental education is effective at reducing AHT events. South Carolina law requires hospitals to offer all new parents with the opportunity to watch an educational video about AHT. This mandate is addressed in different ways at the several delivery centers within a large South Carolina health care system with a range of viewing methods utilized, from DVD players to mobile workstations to personal devices. Frequent technical barriers and workflow inefficiencies resulted in low rates of compliance with this mandate at several campuses. To improve compliance of parent viewing of this educational video, the health care system standardized video viewing protocol across all campuses by implementing the use of iPads for parental education. Existing literature suggests that patient education can be improved in the hospital setting by utilizing tablet computers, but our literature search identified a gap in research around the education of parents and caregivers during hospitalization for childbirth. We used the implementation of an iPad-based parental education delivery protocol to evaluate whether tablet computers can improve compliance with delivering new parent education in the hospital setting.

**Objective:**

The objective of this study was to evaluate whether the standardized use of iPads to deliver education in the mother–baby unit resulted in improved rates of parents’ acceptance of the opportunity to view an educational video about AHT.

**Methods:**

We interviewed physicians and nurses to determine what previous protocols were in place to educate new parents before a standardized iPad-based protocol was implemented across 6 campuses of a large South Carolina health care system. A retrospective study was conducted by review of 5231 records from across the 6 campuses to determine the pre- and postintervention compliance rates of viewing the AHT educational video by parents in the mother–baby unit.

**Results:**

Compliance increased overall (*P*<.001) across sites from an average of 41.93% (SD 46.24) to 99.73% (SD 0.26) (φ=0.510). As much as 4 of 6 locations saw a significant increase in compliance rates after introducing the iPad intervention (*P*<.001). The remaining 2 locations that showed no significant difference (*P*>.05) had very high rates of preintervention compliance.

**Conclusions:**

Following the implementation of a standardized iPad-based protocol to deliver new parent education, there was a significant improvement in the percentage of new parents who viewed an educational video about AHT in the mother–baby unit. Based on these results, other health care providers should consider iPads to be a feasible and effective method for delivering hospital-based education to families in the mother–baby unit.

## Introduction

### Infants and Abusive Head Trauma

Abusive head trauma (AHT) in infants, commonly including “shaken baby syndrome,” is a serious and preventable cause of morbidity and mortality in the first year of life. The Centers for Disease Control and Prevention (CDC) estimates the annual incidence of AHT to be 31-35 per 100,000 infants [1]. Approximately 2000 infants are admitted for AHT to US hospitals annually, with another 1000 annual cases of AHT identified in emergency departments, but not resulting in hospital admissions [2]. These numbers likely underestimated the true incidence of AHT by failing to account for cases that do not result in hospital visits.

### Parent Education Interventions

The American Academy of Pediatrics (AAP) recommends that physicians discuss the risks of AHT with all new parents starting during the birth hospitalization. The AAP specifically recommends that physicians discuss safe ways for parents to cope with the stress of inconsolable infants, provide education on the developmental timeline of crying and other stressful infant behaviors, and the specific dangers of shaking, slamming, or hitting infants [3,4]. CDC recommendations are similar, encouraging providers to ensure that an infant’s parents/caregivers have the coping skills and tools as a preventative measure for AHT for when frustrations arise [5]. Given the increase in stress and residing at home throughout the day with the COVID-19 pandemic, children are more vulnerable than previously [6]. Thus, ensuring patient education of AHT prevention is important now more than ever.

Despite this consensus on the need to provide early parental education on AHT, there is limited evidence from which to base hospital policies and procedures as to exactly what form this education should take or how it should be provided. The United States Preventive Services Task Force performed a systematic analysis concluding that there is a paucity of evidence to determine if interventions in a primary care setting can prevent child abuse and neglect [7]. Nevertheless, limited studies have shown that when health care providers consistently deliver new parent education, there is a long-lasting improvement in parental knowledge concerning AHT [8] that may translate to a reduced local incidence of AHT [9,10]. Existing studies rely on various combinations of educational booklets, DVDs, and signed contracts, with the material provided by nurses or non–health care staff. This education is delivered at varying locations and settings including home visits, in the mother–baby unit, or at prenatal classes. The variable methodology of prevailing studies therefore leaves open the question of which delivery method and media format would best serve to educate new parents on the dangers of AHT.

### Technology and Patient Education

Tablet computers, such as the iPad, are an effective means of easily conveying information to patients in a hospital setting. Prior studies have shown that, when testing patient retention of knowledge, the use of tablets is as effective as verbal education by a nurse [11,12] and superior to education in paper booklets [13]. Patients in the hospital setting also report greater satisfaction [14,15] and higher confidence in their ability to understand information [16] when information is provided via tablet computers, compared with verbal or printed information.

### Legislation and Care of the Newly Born

South Carolina state law requires that all hospitals provide an opportunity for each newborn baby’s parents/caregivers to view a video on the impact of shaking infants and children [17]. It is normal practice for patients in the family beginnings unit and the neonatal intensive care unit to be offered a video on AHT by nurses. The video is shown as part of the 36-hour bundle of care provided to the newborn and family, which includes a series of tests and tasks to be completed 36 hours after birth, such as metabolic, bilirubin, heart, and hearing screening as well as re-weighing of the newborn and viewing AHT and safe sleep education videos. Nurses are expected to set up the video for parents to view, but there is no direct nurse supervision required during video watching.

The law further stipulates that hospitals must ask parents/caregivers to view the video and sign a document attesting that the hospital offered the education [17]. Parents are not required to watch the video, but must sign a state-required form verifying:

I have been offered the opportunity to view the video presentations on safe sleep practices, Sudden Unexpected Infant Death (SUID), and the dangers associated with shaking infants and small children. I have also been given information about the importance of learning infant CPR. I voluntarily sign this statement acknowledging that I have received, read and understood the information and been offered the opportunity to view the videos.

If a parent refuses to watch the video, the nurses assess and document the reason for refusal including if the video was seen during pregnancy or a previous childbirth. At this time nurses also assess if there is a communication deficit such as language barriers or technical difficulties which would not allow for proper viewing of the video.

Hospitals must be compliant with the law and provide an opportunity for video viewing and document the offering of education or reason for refusal. While the video itself is a standardized presentation provided by the state, there is little flexibility in the content of information conveyed, unless a specific request is placed by the hospital system for approval of a different video. Hospitals are, however, given freedom with regard to exactly when, where, and how the video is shown. Thereby an opportunity exists to determine if technology can assist in the delivery of the required parent education. Therefore, the purpose of this paper is to evaluate a quality improvement intervention of delivery of parent education on AHT via iPads within 6 different hospitals within a health care system.

## Methods

### Overview

To improve compliance, defined as parental viewing of the state-mandated video, a quality improvement effort was initiated by digitally converting the video and offering iPads for viewing to standardize information delivery at multiple birth facilities within a large health care system. The implementation was enacted in the same manner at all 6 campuses of the health care system for standardization.

### Original Parent Education Delivery (Method O)

In the past, the video was only available in DVD format (Method O). Barriers to showing the video included inability of nursing staff to locate the DVD and missing or dysfunctional equipment to view the video. The alternative method was to have parents view the video using a workstation on wheels which allowed nurses to access the video in the Care Plan portion of their Epic chart. When this method did not work or was unavailable, the parents were provided a link to view the video on their personal phone, or they would have access to the video from a nurse’s hospital-issued phone.

### iPad Parent Education Delivery (Method N)

Nurses were informed of the new implementation method, reviewed Department of Health and Environmental Control (DHEC) requirements, and discussed administration of the video during staff meetings and “huddles” during their shift. All nursing staff had previous experience with using iPads and did not require training on using the iPads. This intervention was limited to iPads and did not include other tablets. A handout was provided at nursing stations for troubleshooting in the event there were issues with the video or the iPad. The video was also added to the discharge checklist sheet provided to parents to ensure the parents would be able to visualize that the video needed completion before discharge.

Starting in December 2018, communication boards in patient rooms began to include a check off for viewing the required baby education videos. The communication boards were reviewed by the physicians and nurses before discharge as a second check to ensure completion and to attempt to improve compliance.

### Documentation in Electronic Health Record

While the documentation of delivering education in electronic health record (EHR) by nursing staff was unchanged between Method O and Method N, an additional reminder to ensure the educational video was provided was added in the EHR summary tab. In addition to the paper attestation being scanned to the chart, an electronic flowsheet was developed, where nurses documented offering the video and when/whether video was viewed. This improved visibility of the education for physicians and nurses to easily see the workflow during hospitalization. This also allowed reports to be run from the EHR to track compliance rates over time.

### Participants and Procedures

As a University of South Carolina institutional review board–approved retrospective study, data from patients of the 6 campuses were obtained through chart reviews of EHRs to determine newborn educational video compliance. Data were gathered starting in July 2018 and ending in May 2019. However, we found high rates of inconsistent or incomplete documentation during the period of review. The 6 locations yielded 6387 occasions where new parents were offered the AHT video. Events with missing information, such as location, department, date, nurse ID, or compliance were excluded from the study, leaving 5231 records for analysis (comprising 81.90% of data collected). Data for a minimum of 2 weeks prior to the intervention were used to obtain compliance rates before implementation of iPads for each campus, except for campuses 3 and 4, which did not have consistent preintervention data. Data after the intervention were collected for 9 months. The EHR system used by all campuses in the health system was Epic. Discourse with leading stakeholders and implementers of the program provided a platform to gain contextual information regarding the implementation process and barriers of the use of iPads for educational material.

Once a parent or caregiver agreed to watch the educational video, this was documented as “Yes” in the flowsheet tab of the EHR reporting system, which we operationally defined as compliance. The compliance data were extracted from 6 hospitals within the health care system by the internal data support team. Dates of Method N implementation can be found in Table 1.

**Table 1:** Date of iPad education delivery pre- and postintervention by campus location.

Location	Preintervention data collection start date	Implementation date	Postintervention data collection start date
Campus 1	August 1, 2018	August 20, 2018	May 26, 2019
Campus 2	August 1, 2018	August 22, 2018	May 24, 2019
Campus 3	August 1, 2018	August 27, 2018	May 26, 2019
Campus 4	August 1, 2018	October 23, 2018	May 25, 2019
Campus 5	August 1, 2018	October 11, 2018	May 25, 2019
Campus 6	October 27, 2018	November 11, 2018	May 25, 2019

These dates were used as a comparison point to evaluate whether there was an improvement in compliance of parents viewing the video after the implementation of iPads (Method N) compared with Method O. An initial review of the data revealed that a large portion of the extracted data included null values that were evaluated by the data support core. Any portions of the data, for either Method O or Method N, that were incomplete were not included in analysis.

A 1-sided Fisher exact test, a statistical analysis used to assess associations between categorical variables, was performed to determine if the use of iPads to deliver the AHT video significantly increased compliance rates overall and at each of the locations. The Fisher exact test was chosen due to the expected frequency being less than 5 in some of the categories [18]. Discussions with key stakeholders allowed for the identification of barriers and facilitators to the delivery of the video education both before and after implementation.

## Results

EHR data from the 6 locations were assessed to determine compliance rates for viewing the educational AHT video. Results showed that compliance increased overall (*P*<.001) across sites from an average of 41.93% (SD 46.24) to 99.73% (SD 0.26) (φ=.510). Two out of 6 locations saw a significant increase in compliance rates after introducing the iPad intervention (*P*<.001), with 2 campuses showing an increase in compliance that was not statistically significant (*P*>.05). Two campuses showed close to 100% compliance (140/140 [100.00%], 387/389 [99.49%]) after the iPad intervention, but data for compliance rates prior to the intervention were limited. Two sites showed little or incomplete data for the period prior to the intervention, therefore inferential statistics were not conducted for those data (only descriptive). However, those data were included in the overall analyses. For a complete listing of EHR data collection, compliance rates, significance, and effect size, refer to Table 2.

**Table 2:** Fisher exact test results and compliance rates prior to and following the iPad intervention at each location.

Location	% Compliance prior to the intervention (n=632), n/N (%)	% Compliance after the intervention (n=4599), n/N (%)	Significance	Effect size (φ)
Campus 1	24/81 (29.63)	1406/1408 (99.86)	<.001	0.817
Campus 2	5/25 (20.00)	278/279 (99.64)	<.001	0.863
Campus 3	0/86 (0.00)	140/140 (100.00)	N/A^a,b^	N/A
Campus 4	2/92 (2.17)	387/389 (99.49)	N/A	N/A
Campus 5	508/508 (100.00)	1999/1999 (100.00)	>.05	0.022
Campus 6	93/94 (98.94)	389/391 (99.49)	>.05	0.027

^a^N/A: not applicable.

^b^An analysis to compare these with 0 for preintervention could not be performed, because there is not enough variability.

Nurse interviews revealed that, with Method O, there was low compliance because there were not enough TVs to show the video to multiple patients at once, the DVD would go missing, DVD players were not always working in every room, or the remote would get lost or not work. Nurses also reported poor sound quality and volume issues when using the workstation on wheels prior to Method N. Once Method N was implemented with the iPads, the nurses reported minimal issues. If there were technical difficulties with Method N, the nurses had the ability to access the videos using methods previously described in Method O, although no technical difficulties were reported. Nurses and key leaders initiating the intervention reported that the portability of the iPad, along with the decreased challenges that came along with managing multiple equipment components (DVD, player, remote, batteries, TV), has made delivering the educational video easier and quicker.

## Discussion

### Principal Findings

This study was designed to assess the use of iPads in providing AHT prevention education. After the implementation of iPads to provide this educational material, compliance rates increased at all sites, with all nearing 100% after the intervention (Campus 1 1406/1408 [99.86%], Campus 2 278/279 [99.64%], Campus 3 140/140 [100.00%], Campus 4 387/389 [99.49%], Campus 5 1999/1999 [100.00%], and Campus 6 389/391 [99.49%]). Compliance rates of parents watching the video increased from less than 30% to 99% in campuses 1-4 after enacting the iPad intervention. Campuses 5 and 6 demonstrated very high compliance rates before the intervention, and as such, there was no significant difference in compliance rates before and after the intervention for these 2 campuses, with compliance rates remaining near 100% (1999/1999 [100.00%], 389/391 [99.49%]). This study demonstrated that compliance rates for watching the AHT education video were near 100% at all campuses after the implementation of iPads.

Unique aspects of the structure and implementation of the intervention were helpful in conducting this study. Engaging staff in process change decisions has the potential to facilitate early adoption of new patient care methods, and likely resulted in a successful implementation. Additionally, the use of iPads to show patient educational material was a smooth transition due to most staff members being familiar with the use of the device. This allowed each campus to incorporate and edit their respective workflows to suit their campuses the best. A significant hurdle for incorporating changes in hospitals or medical centers is factoring in and attempting to change currently used and long-existing procedures. Workflow interruptions have been found to result in decreased ability of health care providers to manage workload [19]. However, providing smaller changes that are easy to implement and provide flexibility in application have been found to be effective in improving workflow [20]. This implementation of iPads appears to have streamlined the process to decrease workflow barriers, as demonstrated by the increased compliance rates at 4 of the 6 campuses.

The use of iPads to improve delivery of educational material is not a new concept. Specifically, iPads have been useful in the health care setting as a mode of delivering patient education, including education on anticoagulant medication to hospitalized patients, educational materials for patients in acute care, and improvement of inpatients’ engagement in their care and patient education in the hospital [21-23]. Many of these previous studies have shown an improvement in patients’ engagement in their care and education with the use of tablets such as iPads [24,25]. However, little to no research has been conducted on the use of iPads or tablets to increase acceptance of educational material. This study addresses this gap in research and shows that iPads can be used to help increase compliance with patient education delivery.

The compliance with viewing the educational video in the mother–baby unit was widely variable across the 6 sites prior to the implementation of the iPads. Following the intervention, the compliance rates at all sites were near 100%. While we cannot say with certainty that this increase in compliance at most sites was due to the implementation of the iPads, the timing of the increase in compliance rates coincides with the iPad intervention. Although the precise reason for this increase in compliance rates following the intervention remains unclear, a potential theory is that the use of iPads increased ease and efficiency for nurses in their workflow to deliver the educational material, as compared with the original method. Nurses and key leaders in initiating the intervention have reported that the portability of the iPad, along with the advantages of needing less equipment, has made delivering the educational video easier and quicker. To further support this theory, one study demonstrated that nursing staff workflow would allow for delivering a tablet to the patient bedside and retrieving it in a time that is consistent with time between rooms [26].

In our study, all campuses achieved a high compliance rate following the intervention. However, campuses 5 and 6 showed that compliance rates were high both before and after the iPad implementation, as noted previously. Interviews with nurses provide several potential explanations for these unexpected results. At campus 5, a grant had been in place for 4 years to promote further patient engagement with the video in the hospital which provided funding to send each patient home with their own educational DVD, as well as extra training for the staff on the topic of AHT and the importance of patient education. It was reported that during the period of this grant, the compliance rates increased to nearly 90%. This grant ended immediately prior to the implementation of the intervention of the iPads for this study. Thus, nurses and facilitators believed that the pre-existing project focusing on AHT education was the reason for the already high compliance rates of patients watching the video prior to the iPad intervention in campus 5.

Similar results for campus 6 may be explained by its comparatively low delivery volumes. Notably, this campus has a slower pace for the nurses working in the mother–baby unit, according to key leaders who oversaw this intervention. Nursing staff were not pressed for time, which allowed them the ability to provide the educational video regardless of whether it was provided through the TV/DVD method or through the iPad. As such, compliance did not significantly change by incorporating the iPad intervention at this campus. This shows that this intervention may be relatively more effective and necessary in hospitals and medical centers that have a higher workflow, higher patient volume, or are generally busier.

### Limitations and Future Directions

While our study showed an increase in compliance rates after the implementation of the iPads, the results should be viewed in light of the limitations. One of these limitations included missing or null data that were not incorporated in data analysis or results, limiting the overall number of records evaluated. In addition, data collection revealed that 2 of the 6 campuses, campuses 3 and 4, were not accurately recording compliance rates prior to the intervention. This resulted in 18% of the data collected not being included in statistical analysis.

An important consideration is that the periods accessed for the preintervention data were nonstandardized and differed by campus. Some campuses had an exact date of implementation, while others had a week to 2-week range for the date of implementation. While this is not believed to have affected the results significantly, it is still to be considered when looking at the results of this study.

It is important to continue to maintain high compliance of the delivery of this educational material in order to monitor the long-term impact of this intervention. To do this, it is important to monitor nursing staff at regular intervals to ensure delivery of this education via iPads, reassessing workflow to better incorporate this education, ensuring documentation consistency, and involving physicians in the process. Future studies could include assessing documented reasons for refusal, further assessment of nursing workflow by determining the time spent by nursing staff at patient bedside, and the number of interruptions experienced in nursing workflow between the 2 methods. Furthermore, retention of information could be assessed in parents/caregivers who accepted education to determine effectiveness of the education itself, while studies could monitor long-term AHT rates in the community to determine if this education potentially had an impact. Participants’ demographics could also be collected and assessed in future studies to determine if variables such as education level and familiarity with iPads confounded results. Additionally, it is important to note that there is currently insufficient economic data to support the use of digital health interventions in patient education, as noted in current literature [27,28]. While this study did not examine cost-effectiveness of interventions such as this one, future studies should consider whether implementation of iPads for providing patient education could be more cost-effective over time.

Another avenue to look toward is the incorporation of the video in other languages. While the Spanish version of the educational material is currently offered along with the English version, expanding the languages offered and assessing if the appropriate language video affected rate of viewing would also be worthy of study. Perhaps most importantly, alongside long-term use of this intervention, monitoring AHT cases in the geographical areas covered by the health system would be useful to follow to determine if the increased compliance of delivering this education has resulted in a decrease in AHT cases.

### Conclusions

Based on these results, other health care systems may consider iPads or other tablet devices as a feasible and effective method for delivering hospital-based education to patients and families. This intervention has allowed the hospital system to show that they have been compliant with the law as well as meet the guidelines of patient education before discharge.

## Abbreviations

AAP: American Academy of Pediatrics
AHT: abusive head trauma
CDC: Centers for Disease Control and Prevention
CPR: cardiopulmonary resuscitation
DHEC: Department of Health and Environmental Control
EHR: electronic health record
